# Environmental Influences on Sleep: Noise, Lighting, & Social Support in South Asian Caregivers for Older Adults

**DOI:** 10.1093/geroni/igaf122.3095

**Published:** 2025-12-31

**Authors:** Srujana Chekuri, Dinesh Karki, Akankshya Chataut, Julie Blaskewicz Boron

**Affiliations:** University of Nebraska Omaha, Elkhorn, Nebraska, United States; University of Nebraska Omaha, Omaha, Nebraska, United States; University of Nebraska Omaha, Omaha, Nebraska, United States; University of Nebraska Omaha, Omaha, Nebraska, United States

## Abstract

Caregivers for older adults often experience disrupted sleep quality due to environmental stressors, affecting their health, well-being, and caregiving capacity. This mixed-methods study examined how noise, lighting, and social support influence self-reported sleep quality among South Asian caregivers who do not reside with care recipients. Quantitative data were collected from 78 South Asian caregivers (62% male; 38% female), aged 19–73 years (M = 26, SD = 7). Nearly half (48.7%, n = 38) reported frequent noise disturbances, significantly associated with lower sleep quality scores (r= –0.47, p < 0.01). Additionally, 47.4% (n = 37) reported high artificial light exposure before bedtime, linked to slightly lower sleep duration (M = 6.54 hours, SD = 1.59) compared to those with lower exposure (M = 6.78 hours, SD = 1.37), though not statistically significant (t= –0.71, p > 0.05). Higher social support was positively correlated with better sleep quality (r = 0.49, p < 0.001), while 33.3% (n = 26) reported social isolation. Qualitative data from five focus groups (n = 21) were analyzed using inductive thematic analysis. Noise was discussed by 16 participants (76%), including external (e.g., traffic, sirens) and internal (e.g., roommates, appliances) sources, contributing to sleep fragmentation. Lighting was discussed by 14 (67%), who reported screen use and limited daylight as disruptors. Community support, emphasized by 15 (71%), buffered stress and enhanced sleep, while isolation increased anxiety and sleep disruption. Findings highlight the need for culturally responsive environmental and social interventions to improve sleep quality among long-distance caregivers, whose unique challenges, such as physical separation and limited direct support, require targeted strategies to enhance caregiving sustainability and overall well-being.

